# Survey on composition of perennial vegetation in Sesa Mariam Monastery, Northwestern Ethiopia

**DOI:** 10.1186/s13104-015-1562-5

**Published:** 2015-10-30

**Authors:** Birhanu Woldie Meshesha, Berhanu Abraha Tsegay, Birhanu Belay Telake

**Affiliations:** Department of Biology, Debremarkos College of Teacher Education, Debre Marqos, Ethiopia; Department of Biology, Bahir Dar University, Bahir Dar, Ethiopia; Gullelie Botanical Garden, Addis Ababa University, Addis Ababa, Ethiopia

**Keywords:** Diversity, Evenness, Density, Regeneration status, Basal area

## Abstract

**Background:**

Sustainable use of natural resources is one of the leading agenda because anthropogenic activities are leading to the depletion of these resources. Ethiopia is one of the biodiversity reach areas in the world, but the floral diversity is being threatened before they are fully explored. In line with this, very little is known about the flora of Sesa Mariam monastery, found in northwest Ethiopia. The area is one of the few remnant monastery forests in the country with old aged tree species. The aim of the study was to explore and document the floristic composition, density and regeneration status of perennial plant species in order to provide base line information for the sustainable utilization and management of the forest resources.

**Methodology:**

Fifty-one (51) quadrats (20 m × 20 m each) were laid along established transect lines for census of perennial plant species. Two nested quadrats (2 m × 10 m) were also used at the beginning and at the end of every main quadrat for the assessment of seedlings and saplings. All woody plant species in each quadrat were counted and identified. Species diversity, richness and evenness were measured.

**Results:**

One hundred and thirteen (113) plant species belonging to 89 genera and 54 families were identified. Moreover, there were 7 more species outside the study quadrats. Of these plant species 10 were endemic, 92 were indigenous, and the remaining 11 were exotic cultivated trees and shrubs. Fabaceae is the most dominant family with 14 species followed by Euphorbiaceae and Rutaceae, each with 6 species. The total basal area of the matured woody plants of the forest was 94.81 m^2^ ha^−1^ and the density was 1960.78 individuals ha^−1^. The overall diversity and evenness of woody species were 3.81 and 0.85, respectively. When compared to other forests found in Ethiopia, it is better protected.

**Conclusion:**

The data from this study showed a relatively good conservation status. However, analysis from individual woody plant structure, and count of seedlings and saplings showed a need for conservation. Stopping or minimizing grazing by livestock and selective tree cuttings are the first measures to be taken for conservation.

**Electronic supplementary material:**

The online version of this article (doi:10.1186/s13104-015-1562-5) contains supplementary material, which is available to authorized users.

## Background

The flora of Ethiopia is very heterogeneous and it is estimated to contain around 6500–7000 species of higher plants, of which about 12–19 % are endemic [1–4]. This rich diversity and endemism of the country is mainly related with the presence of its diverse ecological features. However, different studies confirmed that all the natural vegetation types of Ethiopia are under harsh threats [5, 6]. According to Alemayehu [7], the speedy depletion of forest resources in Ethiopia has brought significant decline in their biodiversity to the extent that some species are on the verge of local extinction. Because of the early human settlement accompanied by rudimentary farming techniques, extensive cattle husbandry, and ever-increasing human and livestock populations, the rate of deforestation is exceptionally aggravated in the northern Ethiopian highlands [7]. In such devastated areas, conserving and safeguarding of plant diversity has been a very challenging task. Some of the remaining forest patches of Ethiopia are located in the vicinities of churches and monasteries under the protection of the Ethiopian Orthodox Tewahdo Church (EOTC). The EOTC has a long history of planting, protecting and conserving trees. Thus, churches and monasteries are not only considered as religious spots but also as biodiversity hotspot areas.

Sesa Mariam Monastery Forest (SMMF) is one of the remnant forest patches found in northwestern Ethiopia. The dominant plant species of SMMF are more related to Dry Evergreen Montane Forest and recently it was designated as part of the Eastern Afromontane biodiversity hotspots, which made Afromontane rain forests of Ethiopia as one of the 34 hotspot areas in the world [8]. This part of the Afromontane forest is being severely threatened by anthropogenic factors. Therefore, this study was conducted to explore and document the floristic composition, density and regeneration status of the monastery in order to provide base line information for the sustainable utilization and management of the forest resources.

## Methods

### Study area

**Fig. 1:**
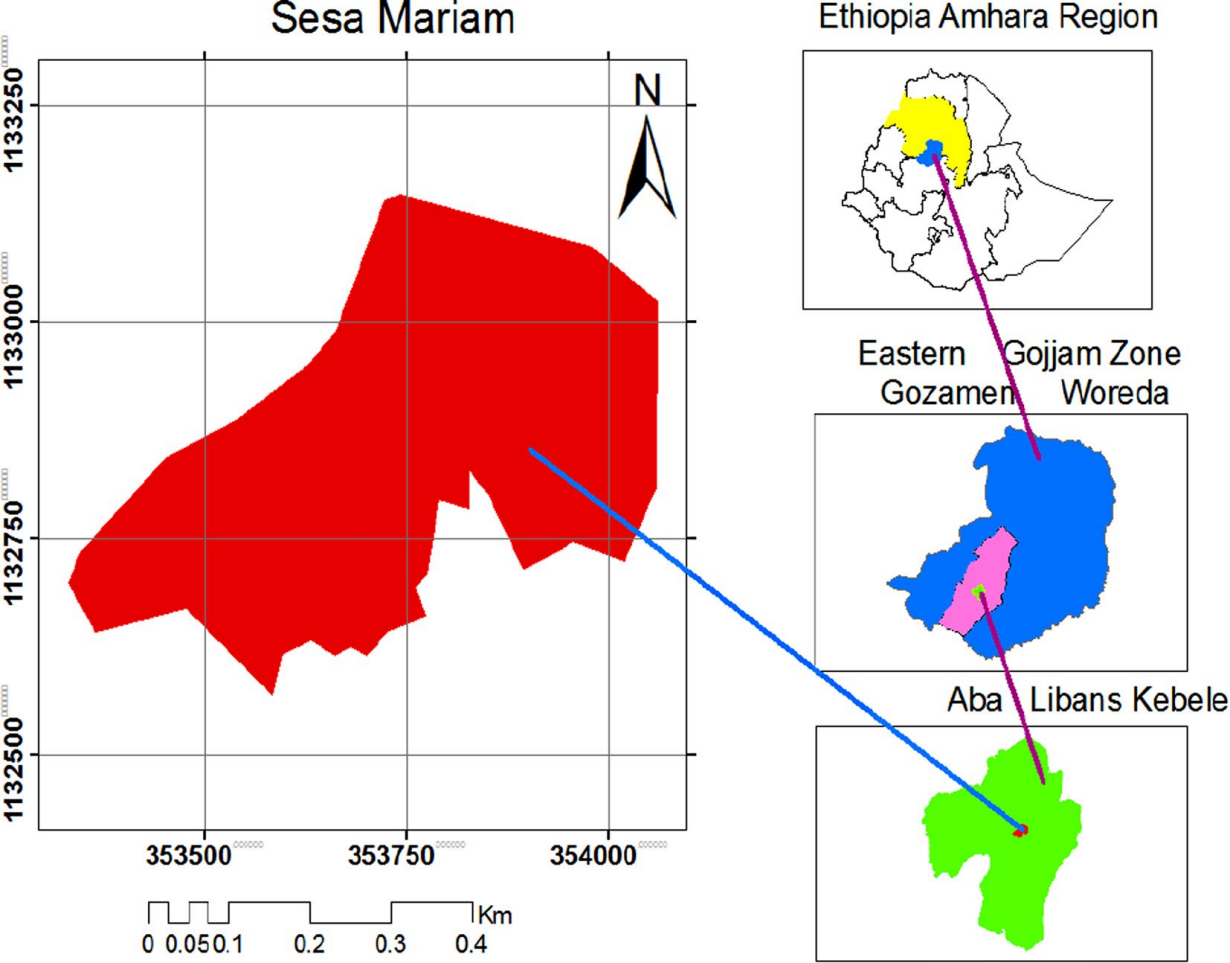
Map of the study area

Sesa Mariam Monastery is found in Gozamin district (locally called Woreda), Northwest Ethiopia. It is located between 10°14′34″N–10°14′53″N latitudes and 37°39′E–37°40′E longitudes (Fig. 1). The altitude ranges between 2090 and 2241 m.a.s.l. The name of the monastery is related to the presence of dominant, big and beautiful trees called *Albizia gummifera* and *Albizia schimperiana*. In the district, the mean annual rain fall ranges between 1448 mm and 1808 mm and the mean annual temperature varies from 11.4 to 25.6 °C. The study area belonged to the Woynadega (mid-temperate mid-highland) agroclimatic zone.

The 2007 population and housing census report of Ethiopia showed that the total population of the district was 116,725. The population size of “Aba Libanos kebele” (localized administrative area), which is the immediate vicinity of the study site, was 7830. From the total 26 “Kebeles” found in the district, this Kebele is the third most crowded area by carrying 233 people per square km. The majority of the population in the district particularly in “Aba Libanos Kebele” depends on rain-fed agriculture, livestock husbandry and irrigation. In addition to the main crops cultivated in the Kebele, timber, charcoal, fire wood and honey products subsidize the local community’s income. Although the monastery lost huge forest lands at different times, dominant and large size tree species like *Prunus africana, Albizia gummifera*, *Albizia schimperiana, Calpurnia aurea, Croton macrostachyus, Juniperus procera*, *Grewia ferruginea*, *Celtis africana* and *Teclea nobilis*; shrubs like *Carissa spinarum* and dominant woody climbers such as *Urera hypselodendron* and *Jasminum abyssinicum* are still intact. It also hosts various species of wild animals including mammals, birds, reptiles and amphibians.

### Methods

Four parallel transects were systematically laid across the forest with an interval of 100 m in the west to east direction. Sample quadrats of 20 m × 20 m were placed along transects at an interval of 50 m. Thus, for the census of mature plants, a total of 51 main quadrats were considered while for the purpose of seedling and sapling inventory, two sub quadrats of 2 m × 10 m were laid at the beginning and the end of each main quadrat. The total seedlings and saplings per quadrat were recorded. In each quadrat, all woody and non-woody plant species were identified by their local names, pressed, coded and then grouped as trees, shrubs, herbs and lianas.

In each quadrat, for all plants having diameter at breast height (DBH) ≥2 cm, the circumference measurements were made at breast height (around 1.3 m) by using measuring tape following the methods described by Martin 1995 [9]. Since stems born from the same root were considered as a single plant during the census of perennial plants, the diameter of stems branching below or at the breast height was measured separately for each branch and summed.

Individual trees and shrubs with DBH <2 cm and height <0.5 m were counted as seedlings while those having DBH <2 cm and height ≥0.5 m were considered as saplings. Perennial plant species that occurred outside the quadrats were also recorded to produce a more complete list of plants but they were not used in subsequent vegetation data analysis. Then, sample plant specimens were properly collected, pressed, dried and carefully identified using the published volumes of Flora of Ethiopia and Eritrea [10–18].

### Data analysis

Matured plant species having DBH ≥2 cm were used in the analysis of vegetation structure. Their abundance, DBH, basal area, density, frequency, and importance value index (IVI) were used in the description of vegetation structure [19]. Species diversity and evenness were measured using the Shannon-Wiener diversity index [19]. To assess the similarity between vegetation samples of the study area and other Ethiopian forests studied with similar methodology, the Sorensen coefficient of similarity index was used [19].

## Results and discussion

### Composition of perennial plants

A total of 113 plant species belonging to 89 genera and 54 families were recorded in all of the 51 quadrats assessed in SMMF (Additional file 1: Appendix). Of these plant species, 55(48.67 %) were trees, 47 (41.59 %) were shrubs and 11 (9.74 %) were woody climbers. Fabaceae is the most abundant family with 14 species followed by Euphorbiaceae and Rutaceae each with 6 species. Celastraceae was represented by 5 species. Poligonacae, Solanacae, Myrtaceae, and Rosaceae were represented each with 4 species while Oleaceae and Tiliaceae each contributed 3 species. Likewise, the remaining 15 families were represented by 2 species each and 29 families each with 1 species. In addition to this, 7 woody plant species were recorded outside the quadrats of the study area. From the total woody species, about 10 were endemic to Ethiopia, 92 were indigenous and 11 were exotic species. The study area contains some national priority tree species which are commercially important. These are *Albizia gummifera*, *Albizia schimperiana, Croton macrostachyus, Celtis africana, Prunus africana* and *Juniperus procera*.

### Species diversity and evenness

Even though the study area is exposed to some anthropogenic activities such as selective tree cutting, grazing and browsing by domestic animals, and mass destruction in some parts, the overall Shannon-Wiener diversity and evenness indices of matured plant species were 3.81 and 0.85, respectively. When these values are compared with the findings of others [7, 20], they are relatively large. These large values indicate that the plant species are diverse and evenly distributed. Such good diversity and evenness is achieved due to the unlimited protective efforts of the Abbot, Monks in the monastery, and some responsible followers of the religion.

### Vegetation structure

Vegetation structure is a very important tool for orienting management activities and assessing the impact of resource extraction [21]. According to Demel [22], vegetation structure is also important to indicate the regeneration status and the past and the present regeneration patterns of the particular vegetation. To display this; density, frequency, diameter at breast height (DBH), basal area (BA) and important value index (IVI) of matured woody plant species were determined.

### Tree density

The total density of woody plant species in all the 51 sample quadrats of the study area was 2230.39 individuals per hectare. The species with the highest density was *Albizia gummifera* (9.55 %) followed by *Justicia schimperiana* (5.38 %), *Carissa spinarum* (4.93 %), *Teclea nobilis* (4.50 %), *Croton macrostachyus* (3.98 %), *Vernonia myriantha* (3.90 %), *Calpurnia aurea* (3.85 %), *Grewia ferruginea* (3.40 %), *Celtis africana* (2.83 %), *Albizia schimperiana* (2.40 %) and *Urera hypselodendron* (2.35 %). These species constituted 47.07 % of all stems in all sampling quadrats of the study area. For the 91 selected woody species, the density distribution was as shown in Table 1.

**Table 1:** Density of matured woody species of the study area at different DBH ranges

DBH (cm)	Density (ha^−1^)	Percentage
2 ≤ DBH < 10	950.00	48.45
10 ≤ DBH ≤ 20	431.86	22.02
>20	578.92	29.53
Total	1960.78	100

The ratio of tree density DBH ≥10 cm and ≤20 cm to DBH >20 cm is taken as a measurement of the size class [23]. The ratio at these densities in this monastery was 0.75. This shows the presence of relatively large differences in abundance between individuals of DBH ≤20 cm and DBH >20 cm. When this value is compared with 13 other natural Afromontane forests found in other parts of Ethiopia, it is very small (Table 2), which confirms the forest is relatively dominated by large plant species.

**Table 2:** Comparison of tree densities with DBH between 10 and 20 (a), and >20 cm (b) of the study area with other forests in Ethiopia arranged in increasing order of a/b values

Forests	(a) 10 cm ≤ DBH ≤ 20 cm	(b) DBH > 20 cm	a/b	Vegetation type	Sources
SMMF	431.86	578.92	0.75	Dry Afromontane	Present study
Egdu (MAM)	155	197	0.79	Dry Afromontane	[24]
Dodola	521	351	1.48	Dry Afromontane	[25]
Wof washa	329	215	1.53	Dry Afromontane	[26]
Gura Lopho	333	210	1.59	Moist Afromontane	[27]
Alata Bolale	365	219	1.67	Moist Afromontane	[28]
Jimma	335	184	1.82	Moist Afromontane	[29]
Denkoro	526	285	1.85	Dry Afromontane	[30]
Gura Ferda	500	263	1.90	Moist Afromontane	[31]
Dindin	437	219	1.99	Dry Afromontane	[6]
Menna Angetu	292.59	139.8	2.09	Moist Afromontane	[32]
Menagesha	484	208	2.33	Dry Afromontane	[26]
Masha Anderacha	385.7	160.5	2.40	Moist Afromontane	[33]
Chilmo	638	250	2.55	Dry Afromontane	[26]

**Fig. 2:**
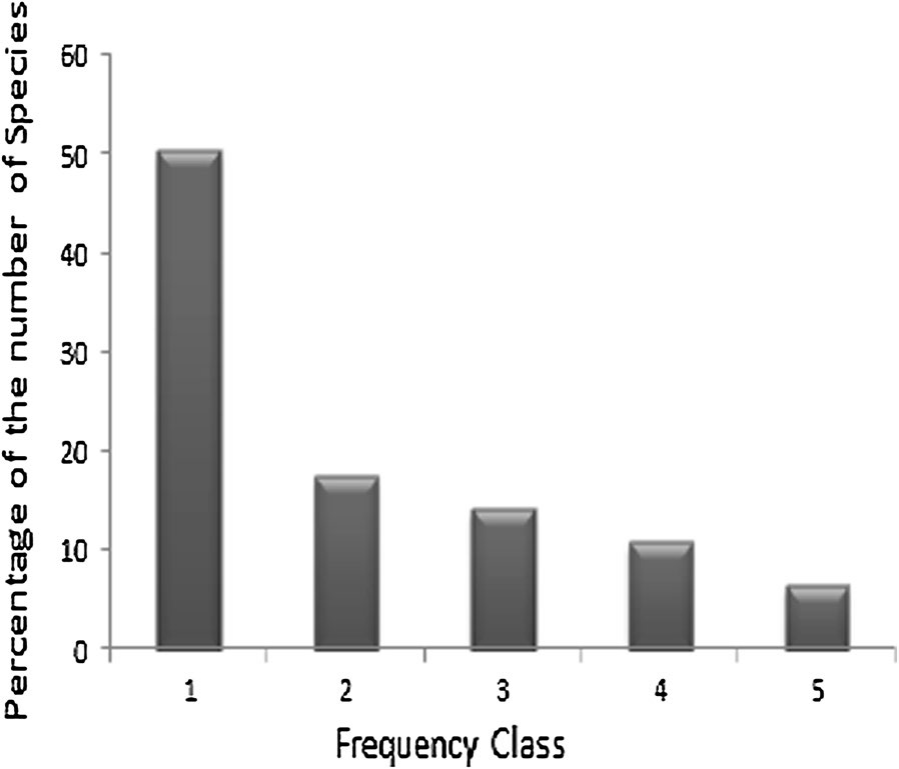
Frequency distribution classes of matured perennial plant species of Sesa Mariam Monastery forest. Key: 1 (0–20 %), 2 (21–40 %), 3 (41–60 %), 4 (61–80 %) and 5 (81–100 %)

Matured plant species of the study area were classified into five frequency classes based on the percentage frequency values (Fig. 2). The present study revealed a progressive decrease in the percentage of the number of species from the lower to the higher frequency classes. This result confirms the existence of high degree of floristic heterogeneity in the study area.

### Diameter at breast height (DBH) distribution

**Fig. 3:**
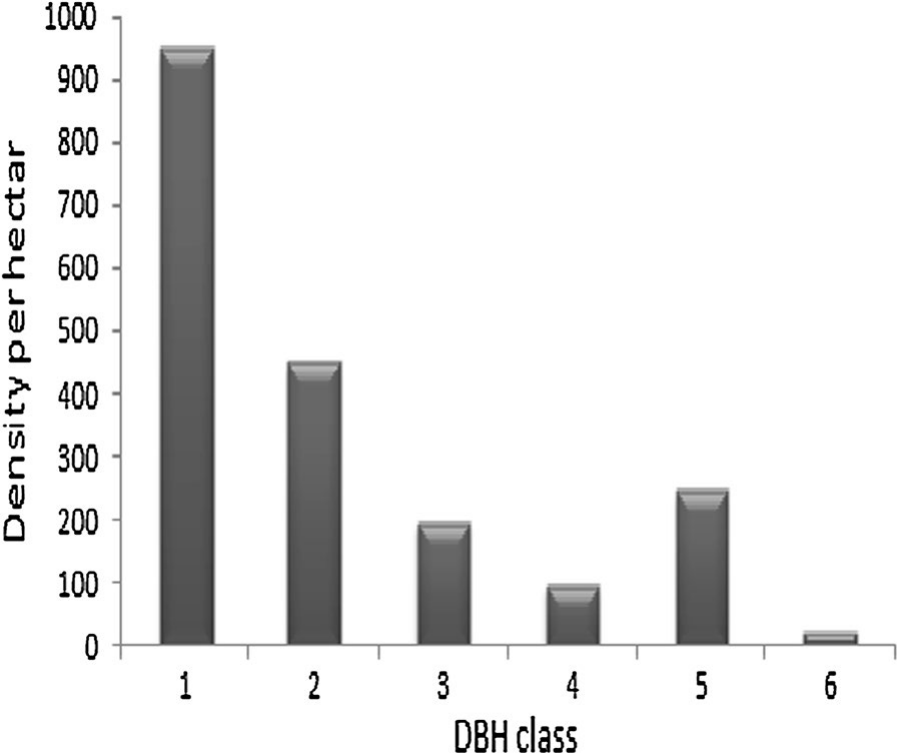
DBH class and density ha^−1^ distribution of matured woody plant species. Key: DBH 1 (2–10.9 cm), 2 (11–20.9 cm), 3 (21–30.9 cm), 4 (31–40.9 cm), 5 (41–50.9 cm) and 6 (≥51 cm) in diameter

The distribution of plant species in different DBH classes is shown in Fig. 3. Matured woody plants of the study area were classified into six DBH classes. The first class had the highest distribution of species density per hectare. With the exception of DBH class 5, the general DBH distribution of trees displayed a decreasing trend from DBH class 1 to DBH class 6. The deviation on DBH class 5 is due to the presence of mature plant species like *Albizia gummifera* and *Albizia schimperiana* whose maximum diameter size is at this class range and they have a relatively better protection from the community for commercial value. About 71.4 % of the tree density ha^−1^ was distributed in the first and second DBH classes but only 1.2 % made the last DBH class. The present study confirmed that not only the existence of a steady decline in density ha^−1^ of individual trees but also the presence of progressive reduction in the number of species from the lower DBH class to the higher DBH class (Fig. 3).

The DBH distribution pattern of woody plant species indicates the general trend of population dynamics and recruitment status of the species [20]. Thus, the DBH distribution of woody plant species of the study area indicates almost an inverted J-curve. This distribution pattern, according to Tamrat [34] and Alemayehu et al. [35], indicates the presence of good regeneration status or stable population structure at the moment in a particular forest.

### Basal area (BA)

The total BA of the study area was 94.81 m^2^ ha^−1^ during study period (Table 3). About 66.86 % of the total BA of the vegetation was covered by *Albizia gummifera, Croton macrostachyus, Albizia schimperiana, Juniperus procera,* and *Prunus africana.* From these, *Albizia gummifera* had the biggest contribution to the BA (42.63 %) of the forest. Measurement of BA provides a good measure of the relative importance of the species than simple stem count. So, species with the largest contribution to the BA can be considered as the most important species in the forest. On the other hand, species like *Justicia schimperiana, Carissa spinarum, Teclea nobilis, Vernonia myriantha,* and *Calpurnia aurea* contributed less to the BA as compared with their densities’ in the forest.

**Table 3:** DBH classes, density ha^−1^, % of density, basal area in m^2^ ha^−1^ and % of basal area of matured woody plants

DBH class (cm)	Density (ha^−1^)	% of density	BA (m^2^ ha^−1^)	% of BA
2–10.9	950	48.45	3.43	3.62
11–20.9	450	22.95	9.52	10.04
21–30.9	195.09	9.95	13.99	14.76
31–40.9	97.06	4.95	11.96	12.61
41–50.9	245.09	12.5	48.27	50.92
≥51	23.53	1.2	7.63	8.05
Total	1960.78	100	94.81	100

Comparison of the basal area density distribution at different DBH classes showed that about 71.4 % of the plants were distributed in the first and second DBH classes which contributed only 13.66 % of basal area, and about 13.7 % were found in the fifth and sixth DBH classes but their contribution for the basal area was 58.97 % (Table 3). This confirmed that the majority of the basal area was not contributed by the occurrence of more number of individuals but by the presence of some most important plant species of the forest.

The highest BA value recorded in DBH class five was due to the higher contribution of *Albizia gummifera* both in abundance and diameter. When the study area is compared with 15 other monastery and natural forests, it has smaller value than three areas (Table 4). This may be due to the difference in geographical location and the age of the forests but not related with the lack of conservation effort.

**Table 4:** Basal area comparisons of Sesa Mariam monastery forest with 15 other Afromontane monasteries and natural forests found in different parts of the Ethiopia

Forests	BA (m^2^ ha^−1^)	Vegetation type	Sources
Dodola	129	Dry Afromontane	[25]
Tara Gedam	115.36	Dry Afromontane	[20]
Wof Washa	101.80	Dry Afromontane	[26]
SMMF	94.81	Dry Afromontane	Present study
Menna Angetu	94.22	Moist Afromontane	[32]
Egdu (MAM)	84.17	Dry Afromontane	[24]
Masha Anderacha	81.90	Moist Afromontane	[33]
Gura Ferda	69.90	Moist Afromontane	[31]
Alata Bolale	53.33	Moist Afromontane	[28]
Abebaye forest	49.45	Dry Afromontane	[20]
Dindin	49	Dry Afromontane	[6]
Denkoro	45	Dry Afromontane	[30]
Menagesha	36.10	Dry Afromontane	[26]
Jimma	33.30	Moist Afromontane	[29]
Chilmo	30.10	Dry Afromontane	[26]
Gura Lopho	29.63	Moist Afromontane	[27]

### Importance value index (IVI)

Values of IVI are important parameters that reveal the ecological significance of species in a particular ecosystem [36]. Moreover, species with the highest IVI values are the most dominant of the particular vegetation [6]. The analysis of IVI values of the study area revealed that the forest is dominated by few woody plant species like *Albizia gummifera*. The dominant plant species in the study area (descending order of IVI) were *Albizia gummifera, Croton macrostachyus, Albizia schimperiana, Juniperus procera, Calpurnia aurea, Teclea nobilis*, Vernonia myriantha*, Celtis africana, Justicia schimperiana, Carissa spinarum, Prunus africana, Cordia africana, Urera hypselodendron,* and *Grewia ferruginea.* These species contributed about 56 % of the total IVI. The highest IVI of *Albizia gummifera* was due to its high values of relative frequency, relative dominance and relative density. Important value indices are good indicators for prioritization of species conservation [6].

### Regeneration status of plants in Sesa Mariam Monstry Forest

The composition, distribution, and density of seedlings and saplings indicate the future appearance of a particular forest. Thus, regeneration or recruitment potential of plants is one of the major factors that are useful to assess their conservation status. From the entire 113 plants in the Monastry, a total of 849.52 seedlings ha^−1^, 1211.77 saplings ha^−1^, and 1960.78 matured plants ha^−1^ were recorded and identified. The ratio of seedlings and saplings to the matured woody plants was 0.43 and 0.62, respectively. These results indicated the presence of lower number of seedlings and saplings than the matured ones. This could be due to human intervention, browsing and grazing effects, shade effect, soil erosion and the presence of pests which attack seeds, seedlings and/or saplings. Seedling and sapling abundance analysis confirmed that the regeneration potential of the forest may be in problem unless proper management strategies are soon carried out. Thus, to indicate the conservation priority, woody plant species of the study area were classified into three classes based on their abundance [37]. The first group contained those plant species that do not have seedlings at all and those that are represented by only one seedling. Plant species having seedling abundance >1 but <5 individuals belonged in the second group and the third group comprised of those having seedling individuals of ≥5. From these, group 1 and group 2 require priority for conservation. Besides, appropriate conservation measures should be given for the third group in order to retain the diversity of woody plant species for the far future.

### Phytogeographical comparison

Sorensen’s similarity coefficient index was used to compare woody plant composition of Sesa Mariam Monastery forest with 10 Afromontane forests found in different parts of Ethiopia (Table 5).

**Table 5:** Comparison of species similarity between Sesa mariam Monastry forest and other different Ethiopian Afromontane forests as calculated by Sorensen’s similarity index

Forests	Altitude (m.a.s.l.)	Latitude (N)	Longitude (E)	A	B	C	Sc (%)	Sources
Sesa Mariam	2090–2241	10°14′–10°14′	37°39′–37°40′	–	–	–	–	
Gedo	1300–3060	09°01′–09°02′	37°16′–37°25′	61	59	38	55.71	[38]
Tara Gedam	2142–2484	12°06′–12°07′	37°46′–37°47′	57	63	42	52.05	[20]
Abebaye	1921–2072	12°06′–12°07′	37°46′–37°47′	50	70	28	50.51	[20]
Gura Lopho	2154–2364	09°56′–09°57′	36°58′–37°01′	47	73	51	43.12	[27]
Zegie Peninsula	1770–1975	11°40′–11°43′	37°19′–37°21′	50	70	63	42.92	[39]
Adey Amba	2090–2603	12°31′–12°33′	37°39′–37°41′	34	86	16	40.00	[40]
Sese	1476–1780	08°28′–08°29′	35°45′–35°50′	43	77	64	37.89	[41]
Mana-Angetu	1533–2431	06°10′–06°31′	39°30′–39°45′	43	77	106	31.97	[32]
Gurra-Ferda	1650–2055	06°45′–07°08′	35°00′–35°15′	29	91	37	31.18	[31]
Denkoro	2300–3400	10°35′–11°15′	38°30′–39°07′	27	93	38	29.19	[30]

Total number of plant species in Sesa mariam Forest (SMMF) = 113

Sc (Sorensen’s similarity coefficient) = 2A/2A + B + C

*A* plant species common to SMMF and the forest in comparison, *B* plant species found only in SMMF, *C* plant species found only in the forest in comparison with SMMF

Based on the information from Table 5, the study area shared the highest similarity with Gedo Dry Afromontane forest (55.71 %) followed by Tara Gedam (52.05 %) and Abebaye forest (50.51 %). This might be due to similarities in temperature, rain fall and altitudinal ranges.

## Conclusion

It is obvious that the monastery has lost extensive forestlands at various times in the past. However, currently the church community has recognized the importance of the remaining forest to its existence and the ecosystem. Hence, it has made the survival of the diversified indigenous plants possible. Regardless of such positive attitude from the church community and the relative conservation efforts made, seedling and sapling abundance analysis revealed 71 out of 113 woody plant species to be represented by either one or no seedlings at all. This shows that the regeneration potential of the forest is declining and intervention measures are needed. Grazing by livestock and farm land expansion are the major threats to the forest resources, which demands a necessity for more concerted effort towards protection of the church forest.

## Additional file

10.1186/s13104-015-1562-5 List of plant species in Sesa Mariam monastery forest showing the total basal area in m^2^ha^−1^(BA in M^2^Ha^−1^), density ha^−1^, relative frequency (RF), relative density (RD), relative dominance (RDo), importance value index (IVI), and percent of importance value index (%IVI) in descending order of priority classes (PC).

## Abbreviations

SMMF: Sesa Mariam Monastery Forest
USAID: United States Agency for International Development
EOTC: Ethiopian Orthodox Tewahdo Church
DBH: Diameter at breast height
BA: Basal area
IVI: Importance value index
SC: Sorenson’s similarity Coefficient
