# Supplementary material for: Survey on composition of perennial vegetation in Sesa Mariam Monastery, Northwestern Ethiopia
Source: BMC Res Notes. 2015 Oct 30;8:622. doi: 10.1186/s13104-015-1562-5 (PMC4627382; doi:10.1186/s13104-015-1562-5)
Supplement: Supplementary file 1 — 10.1186/s13104-015-1562-5 List of plant species in Sesa Mariam monastery forest showing the total basal area in m2ha−1(BA in M2Ha−1), density ha−1, relative frequency (RF), relative density (RD), relative dominance (RDo), importance value index (IVI), and percent of importance value index (%IVI) in descending order of priority classes (PC). [file 13104_2015_1562_MOESM1_ESM.docx]

Additional file 1 (Appendix)

List of plant species in Sesa Mariam monastery forest showing the total basal area in m^2^ha^-1^(BA in M^2^Ha-^1^), density ha^-1^, relative frequency (RF), relative density (RD), relative dominance (RDo), importance value index (IVI), and percent of importance value index (%IVI) in descending order of priority classes (PC).

| Species name | Family | Life form | BA in M^2^Ha-^1^ | Density Ha^-1^ | RF | RD | Rdo | IVI | %IVI | PC^*^ |
| --- | --- | --- | --- | --- | --- | --- | --- | --- | --- | --- |
| *Albizia gummifera* (J. F. Gmel.) C.A. Sm. | Fabaceae | T | 40.42 | 187.25 | 3.52 | 9.55 | 42.60 | 55.70 | 18.57 | 4 |
| *Albizia schimperiana* Oliv. | Fabaceae | T | 5.59 | 47.06 | 2.15 | 2.40 | 5.89 | 10.44 | 3.48 | 3 |
| *Croton macrostachyus* Del. | Euphorbiaceae | T | 6.78 | 77.94 | 3.3 | 3.98 | 7.16 | 14.4 | 4.81 | 3 |
| *Allophylus abyssinicus* (Hochst.) Radlkofer | Sapindaceae | T | 0.79 | 36.76 | 2.01 | 1.88 | 0.83 | 4.72 | 1.57 | 2 |
| *Bersama abyssinica* Fresen.*subsp.abyssinica* | Melianthaceae | T | 1.0 | 38.24 | 2.01 | 1.95 | 1.06 | 5.02 | 1.67 | 2 |
| *Brucea antidysenterica* J. *F. Mill.* | Simaroubaceae | T | 0.04 | 33.33 | 2.94 | 1.70 | 0.04 | 4.68 | 1.56 | 2 |
| *Buddleja polystachya* Fresen. | Loganiaceae | S/T | 0.05 | 38.24 | 2.94 | 1.95 | 0.05 | 4.94 | 1.65 | 2 |
| *Calpurnia aurea* | Fabaceae | S/T | 1.55 | 75.49 | 3.45 | 3.85 | 1.63 | 8.93 | 2.98 | 2 |
| *Clausena anisata*(Willd.) Benth. | Rutaceae | S | 0.17 | 34.8 | 2.29 | 1.78 | 0.18 | 4.25 | 1.42 | 2 |
| *Capparis tomentosa* Lam. | Capparidaceae | C | 0.14 | 42.65 | 2.15 | 2.18 | 0.15 | 4.48 | 1.49 | 2 |
| *Carissa spinarum* L. | Apocynaceae | S | 0.32 | 96.57 | 2.58 | 4.93 | 0.33 | 7.84 | 2.61 | 2 |
| *Celtis africana Burm. f.* | Ulmaceae | S/T | 3.88 | 55.39 | 1.29 | 2.83 | 4.09 | 8.21 | 2.74 | 2 |
| *Cordia africana* Lam. | Boraginaceae | T | 3.87 | 28.92 | 1.22 | 1.48 | 4.08 | 6.78 | 2.26 | 2 |
| *Cupressus lusitanica* Mill. | Cupressaceae | T | 1.61 | 32.35 | 0.65 | 1.65 | 1.69 | 3.99 | 1.33 | 2 |
| *Dombeya torrid* subsp. torriad | Sterculiaceae | S/T | 0.64 | 32.84 | 1.44 | 1.68 | 0.67 | 3.79 | 1.26 | 2 |
| *Dracaena steudneri Engl.* | Dracaenaceae | T | 1.08 | 17.16 | 1.51 | 0.88 | 1.14 | 3.53 | 1.18 | 2 |
| *Grewia ferruginea* Hochst. ex A. Rich. | Tiliaceae | S | 0.31 | 66.67 | 2.79 | 3.40 | 0.32 | 6.51 | 2.17 | 2 |
| *Jasminum grandiflorum L.* | Oleaceae | T | 0.05 | 26.96 | 1.72 | 1.38 | 0.05 | 3.15 | 1.05 | 2 |
| *Juniperus procera* Hochst. ex Endl. | Cupressaceae | T | 5.35 | 43.63 | 2.01 | 2.23 | 5.64 | 9.88 | 3.29 | 2 |
| *Justicia schimperiana* (Hochst. ex Nees) T. Anders. | Acanthaceae | S | 0.07 | 105.39 | 2.51 | 5.38 | 0.08 | 7.97 | 2.66 | 2 |
| *Maytenus obscura* (A. Rich.) Cuf. | Celastraceae | S | 0.11 | 40.19 | 2.79 | 2.05 | 0.11 | 4.95 | 1.65 | 2 |
| *Nuxia congesta KBr.* ex *Fresen.* | Loganiaceae | S/T | 1.05 | 30.88 | 1.01 | 1.58 | 1.11 | 3.70 | 1.23 | 2 |
| *Olea europaea subsp. Cuspidata* (Wall. ex G. Don) Cif. | Oleaceae | T | 2.09 | 9.8 | 1.15 | 0.50 | 2.20 | 3.85 | 1.28 | 2 |
| *Olinia rochetiana* A. Juss. | Oliniaceae | S/T | 0.26 | 34.31 | 1.94 | 1.75 | 0.28 | 3.97 | 1.32 | 2 |
| *Premna schimperi Engl.* | Lamiaceae | S/T | 0.43 | 39.71 | 2.29 | 2.03 | 0.45 | 4.77 | 1.59 | 2 |
| *Prunus africana*(Hook. f.) Kalkm. | Rosaceae | T | 5.25 | 12.25 | 1.51 | 0.63 | 5.53 | 7.67 | 2.56 | 2 |
| *Pterollobium stellatum*(Forssk.) Brenan | Fabaceae | C | 0.17 | 34.31 | 2.44 | 1.75 | 0.18 | 4.37 | 1.46 | 2 |
| *Rosa abyssinica* Lindley | Rosaceae | S/C | 0.08 | 37.75 | 2.15 | 1.93 | 0.08 | 4.16 | 1.39 | 2 |
| *Senna singueana*(Del.) Lock | Fabaceae | S/T | 0.17 | 34.8 | 2.51 | 1.78 | 0.19 | 4.48 | 1.49 | 2 |
| *Teclea nobilis Del.* | Rutaceae | S/T | 0.81 | 88.24 | 3.45 | 4.50 | 0.86 | 8.81 | 2.94 | 2 |
| *Urera hypselodendron* (A. Rich.) Wedd. | Urticaceae | C | 1.04 | 46.08 | 3.16 | 2.35 | 1.09 | 6.60 | 2.20 | 2 |
| Vernonia myriantha *Hook.f* | Asteraceae | S/T | 1.05 | 76.47 | 3.23 | 3.90 | 1.11 | 8.24 | 2.75 | 2 |
| Esat alash | Unidentified | T | 0.66 | 23.04 | 1.08 | 1.18 | 0.69 | 2.95 | 0.98 | 1 |
| *Acacia abyssinica*  Hochst. ex Benth. | Fabaceae | T | 0.47 | 1.96 | 0.29 | 0.10 | 0.50 | 0.89 | 0.29 | 1 |
| *Acacia brevispica Harms* | Fabaceae | S | 0.43 | 4.9 | 0.50 | 0.25 | 0.46 | 1.21 | 0.40 | 1 |
| *Acacia lahai* Steud. & Hochst. ex Benth. | Fabaceae | T | 0.52 | 15.69 | 0.79 | 0.80 | 0.55 | 2.14 | 0.71 | 1 |
| *Acacia polyacantha subsp.campylacantha* (Hochst. ex A. Rich.) Brenan | Fabaceae | T | 0.12 | 2.94 | 0.43 | 0.15 | 0.13 | 0.71 | 0.24 | 1 |
| *Acacia seyal* Del. | Fabaceae | T | 0.4 | 3.92 | 0.36 | 0.20 | 0.43 | 0.99 | 0.33 | 1 |
| *Acalypha fruticosa Forssk.* | Tiliaceae | S | 0.01 | 5.88 | 0.50 | 0.30 | 0.01 | 0.81 | 0.27 | 1 |
| *Acalypha psilostachya Hochst.* | Euphorbiaceae | S | 0.02 | 13.73 | 0.65 | 0.70 | 0.02 | 1.37 | 0.46 | 1 |
| *Acokanthera schimperi* (A. *DC.) Schweinf.* | Apocynaceae | S/T | 0.05 | 1.47 | 0.14 | 0.08 | 0.05 | 0.27 | 0.09 | 1 |
| *Adansonia digitata L.* | Bambacaceae | T | 0.07 | 2.94 | 0.36 | 0.15 | 0.08 | 0.59 | 0.19 | 1 |
| *Argemone mexicana L.* | Papaveraceae | S | 0.01 | 7.84 | 0.29 | 0.40 | 0.01 | 0.7 | 0.23 | 1 |
| *Arundo donax L.* | Poaceae | S | 0.01 | 4.9 | 0.07 | 0.25 | 0.01 | 0.33 | 0.11 | 1 |
| *Chenopodium album L.* | Chenopodiaceae | S | 0.01 | 2.45 | 0.14 | 0.13 | 0.01 | 0.28 | 0.09 | 1 |
| *Carica papaya* L. | Caricaceae | T | 0.01 | 0.98 | 0.07 | 0.05 | 0.01 | 0.13 | 0.04 | 1 |
| *Citrus medica L.* | Rutaceae | T | 0.02 | 0.98 | 0.07 | 0.05 | 0.02 | 0.14 | 0.05 | 1 |
| *Citrus aurantifolia (Christm.) Swingle* | Rutaceae | T | 0.01 | 0.49 | 0.07 | 0.03 | 0.01 | 0.11 | 0.04 | 1 |
| *Citrus sinensis (L.) Osb.* | Rutaceae | T | 0.01 | 1.47 | 0.07 | 0.08 | 0.01 | 0.16 | 0.05 | 1 |
| *Clerodendrum myricoides (Hochst.) Vatke* | Lamiaceae | S | 0.06 | 17.16 | 1.79 | 0.88 | 0.06 | 2.73 | 0.91 | 1 |
| *Coffea Arabica* L. | Rubiaceae | S | 0.01 | 0.98 | 0.07 | 0.05 | 0.01 | 0.13 | 0.04 | 1 |
| *Delphinium dasycaulon Fresen.* | Ranunculaceae | T | 0.06 | 0.98 | 0.14 | 0.05 | 0.07 | 0.26 | 0.09 | 1 |
| *Dichrostachys cinerea* (L.) Wight &Arn. | Fabaceae | S | 0.02 | 10.29 | 1.15 | 0.53 | 0.02 | 1.70 | 0.57 | 1 |
| *Dodonaea angustifolia L. f.* | Sapindaceae | S | 0.01 | 1.96 | 0.07 | 0.10 | 0.01 | 0.18 | 0.06 | 1 |
| *Ensete ventricosum* (Welw.) Cheesman | Musaceae | H | 0.01 | 2.45 | 0.14 | 0.13 | 0.01 | 0.28 | 0.09 | 1 |
| *Erythrina abyssinica Lam. exDC.* | Fabaceae | T | 1.07 | 9.31 | 1.08 | 0.48 | 1.13 | 2.69 | 0.89 | 1 |
| *Erythrina brucei Schwein f.* | Fabaceae | T | 0.29 | 4.9 | 0.72 | 0.25 | 0.31 | 1.28 | 0.43 | 1 |
| *Eucalyptus comaldulensis* | Myrtaceae | T | 0.05 | 1.96 | 0.07 | 0.10 | 0.05 | 0.22 | 0.07 | 1 |
| *Eucalyptus globulus* | Myrtaceae | T | 0.18 | 0.98 | 0.14 | 0.05 | 0.19 | 0.38 | 0.13 | 1 |
| *Euclea racemosa subsp.schimperi (A. DC.)* White | Eubenaceae | S | 0.03 | 24.02 | 1.36 | 1.23 | 0.03 | 2.62 | 0.87 | 1 |
| *Euphorbia abyssinica Gmel.* | Euphorbiaceae | T | 0.37 | 14.71 | 0.29 | 0.75 | 0.39 | 1.43 | 0.48 | 1 |
| *Ficus sur Forssk.* | Moraceae | T | 1.39 | 6.86 | 0.36 | 0.35 | 1.47 | 2.18 | 0.73 | 1 |
| *Ficus vasta Forssk.* | Moraceae | T | 0.37 | 0.98 | 0.14 | 0.05 | 0.39 | 0.58 | 0.19 | 1 |
| *Gnidia glauca* (Fresen.) Gilg | Thymelacaceae | S/T | 0.09 | 2.45 | 0.22 | 0.13 | 0.09 | 0.44 | 0.15 | 1 |
| *Grewia bicolor Juss.* | Tiliaceae | S/T | 0.01 | 1.47 | 0.07 | 0.08 | 0.01 | 0.16 | 0.05 | 1 |
| *Impatiens ethiopica* Grey-Wilson | Balsaminaceae | H | 0.01 | 8.33 | 0.79 | 0.43 | 0.01 | 1.23 | 0.41 | 1 |
| *Impatiens rothii* Hook. f. | Balsaminaceae | H | 0.01 | 11.76 | 1.15 | 0.6 | 0.01 | 1.76 | 0.59 | 1 |
| *Kalanchoe. Spp.* | Crassulaceae | S | 0.01 | 3.92 | 0.29 | 0.20 | 0.01 | 0.50 | 0.17 | 1 |
| *Mangifera indica* L. | Anacardiaceae | T | 0.04 | 0.49 | 0.07 | 0.03 | 0.04 | 0.14 | 0.05 | 1 |
| *Maytenus arbutifolia (A. Rich.) Wilczek* | Celastraceae | S | 0.09 | 14.71 | 1.79 | 0.75 | 0.10 | 2.64 | 0.88 | 1 |
| *Millettia ferruginea (Hochst.) Bak.* | Fabaceae | T | 0.03 | 12.26 | 1.44 | 0.63 | 0.03 | 2.10 | 0.7 | 1 |
| *Mimusops kummel* A. *DC.* | Sapotaceae | T | 0.14 | 1.47 | 0.14 | 0.08 | 0.15 | 0.37 | 0.12 | 1 |
| *Musa x paradisiaca L.* | Musaceae | H | 0.01 | 0.98 | 0.07 | 0.05 | 0.01 | 0.13 | 0.04 | 1 |
| *Olea capensis L. subsp. macrocarpa* (C. H. Wright)Verdc. | Oleaceae | T | 0.14 | 2.45 | 0.29 | 0.13 | 0.14 | 0.56 | 0.19 | 1 |
| *Osyris quadripartita* Decn. | Santalaceae | T | 0.06 | 17.16 | 1.36 | 0.88 | 0.06 | 2.30 | 0.77 | 1 |
| *Pavetta abyssinica* | Rubiaceae | S/T | 0.13 | 21.08 | 1.51 | 1.08 | 0.14 | 2.73 | 0.91 | 1 |
| *Persea americana* Mill. | Lauraceae | T | 0.01 | 1.47 | 0.07 | 0.08 | 0.01 | 0.16 | 0.05 | 1 |
| *Phoenix reclinata Jacq.* | Arecaceae | T | 0.01 | 0.49 | 0.07 | 0.03 | 0.01 | 0.11 | 0.04 | 1 |
| *Psidium guajava L.* | Myrtaceae | T | 0.01 | 0.98 | 0.07 | 0.05 | 0.01 | 0.13 | 0.04 | 1 |
| *Pittosporum viridiflorum* Sims | Pittosporaceae | S | 0.04 | 0.98 | 0.07 | 0.05 | 0.05 | 0.17 | 0.06 | 1 |
| *Prunus persica (L.) Batsch* | Rosaceae | T | 0.02 | 1.47 | 0.14 | 0.08 | 0.03 | 0.25 | 0.08 | 1 |
| *Rhamnus prinoides L'Herit.* | Rhamnaceae | S/T | 0.01 | 1.47 | 0.07 | 0.08 | 0.01 | 0.16 | 0.05 | 1 |
| *Rhamnus staddo* A. Rich.  *var.deflersii* (Schweinf. ex Herzog) Chiov. ex Engl. | Rhamnaceae | S/T | 0.02 | 0.98 | 0.14 | 0.05 | 0.02 | 0.21 | 0.07 | 1 |
| *Rhus natalensis* Krauss | Anacardiaceae | S/T | 0.08 | 6.37 | 0.36 | 0.33 | 0.08 | 0.77 | 0.26 | 1 |
| *Ricinus communis* L. | Euphorbiaceae | H | 0.01 | 1.47 | 0.07 | 0.08 | 0.01 | 0.16 | 0.05 | 1 |
| *Rubus apetalus Poir.* | Rosaceae | S | 0.01 | 7.35 | 1.08 | 0.38 | 0.01 | 1.47 | 0.49 | 1 |
| *Syzygium guineense (Willd.) DC.* | Myrtaceae | T | 0.79 | 5.88 | 0.57 | 0.3 | 0.83 | 1.70 | 0.57 | 1 |
| *Urtica simensis Steudel* | Urticaceae | H | 0.01 | 22.06 | 0.50 | 1.13 | 0.02 | 1.65 | 0.55 | 1 |
| *Vernonia amygdalina Del.* | Asteraceae | S/T | 0.02 | 3.43 | 0.50 | 0.18 | 0.03 | 0.71 | 0.24 | 1 |
| *Vernonia auriculifera Hiern.* | Asteraceae | S/T | 0.09 | 11.76 | 1.44 | 0.60 | 0.09 | 2.13 | 0.71 | 1 |
| *Ximenia americana L.* | Olacaceae | S/T | 0.01 | 0.49 | 0.07 | 0.03 | 0.01 | 0.11 | 0.04 | 1 |
| Total |  |  | 94.81 | 1960.78 | 100 | 100 | 100 | 300 | 100 |  |

PC^* -^  priority is based on abundance/regeneration capacity
